# Daptomycin for treatment of methicillin-resistant Staphylococcus epidermidis saphenectomy wound infection after coronary artery bypass graft operation (CABG): a case report

**DOI:** 10.1186/1749-8090-4-47

**Published:** 2009-09-11

**Authors:** Jan D Schmitto, Aron F Popov, Samuel T Sossalla, Kasim O Coskun, Suyog A Mokashi, Anton Wintner, Friedrich A Schoendube

**Affiliations:** 1 Department of Thoracic-, Cardiac- and Vascular Surgery, Georg-August-University of Goettingen, Goettingen, Germany; 2 Department of Cardiology, Georg-August-University of Goettingen, Goettingen, Germany; 3 Division of Cardiac Surgery, Brigham and Women's Hospital, Harvard Medical School Boston, MA, USA

## Abstract

We report a case of successful treatment of postoperative saphenectomy wound infection of the upper left leg with the antibiotic drug Daptomycin.

## Introduction

Since its first clinical use by Rene Favaloro in the 60's, the great saphenous vein has become the most commonly harvested conduit for revascularization in coronary artery bypass grafting (CABG) [1]. In order to reduce morbidity and improve the recovery time associated with CABG procedures, various techniques have been developed including conventional conduit harvesting, minimally invasive and/or endoscopic harvesting procedures [2]. Still, these surgical techniques are associated with significant complication rates e.g. wound infections, non-infective wound healing disturbances, postoperative pain, etc. [3]. Avoiding and/or reducing these complication rates is of great medical and economic interest. Improvements would result in increased postoperative mobility and quality of life as well as reduced length of hospital stay following surgery resulting large cost savings. Although much research has focused on comparing less invasive and conventional harvest techniques, there is at present no consensus on the areas of postoperative antibiotic drug treatment of saphenectomy wound infections once an infection occurs. Further studies are required to compare treatment methods of saphenectomy wound infections by different antibiotic drugs. Although, Daptomycin has already been proven to be effective in the treatment of bacteremia and endocarditis [4,5] caused by methilin-resistent Staphylococcus aureus [6,7] and several case reports about its effectiveness in the field of cardiac surgery exist in the literature [8], there are still no cases describing the successful treatment of saphenectomy wound infections by Daptomycin.

Therefore, in this paper we report the first case of successful treatment of a postoperative wound infection after saphenectomy of the great saphenous vein of the upper left leg with the new antibiotic drug Daptomycin (Cubicin^®^, Novartis Pharma Corporation, Germany).

## Case report

We report the case of a 68-year old man with severe, diffuse coronary artery disease who presented with two months of progressive angina-pectoris-symptoms. Coronary angiography verified 3-vessel-disease with significant stenoses of the main coronary branches (LAD 99%, RCX 80%, RCA 90%), moderately-decreased systolic left-ventricular function and ST-elevations in leads II, III and aVF. For these reasons, surgical revascularization was indicated. The patient had a significant history of arterial hypertension, Type II diabetes mellitus, adipositas, nicotine abuse (49 pack years), hypercholersterinemia and performance of PTCA of the left coronary artery in 2002.

In the Department of Thoracic-, Cardiac- and Vascular Surgery of the University of Goettingen, Germany, we performed CABG with three single vein-grafts to the diagonal branch (DB), right posterior descending artery (RPD) and left posterolateral circumflex artery (LPL). The left anterior descending artery (LAD) was revascularized by the left internal mammarian artery (LIMA). Harvest of the saphenous vein was performed by conventional open saphenectomy harvest technique which involved an incision extending from the left medial malleolus, along the medial aspect of the knee to the groin.

**Figure 1:**
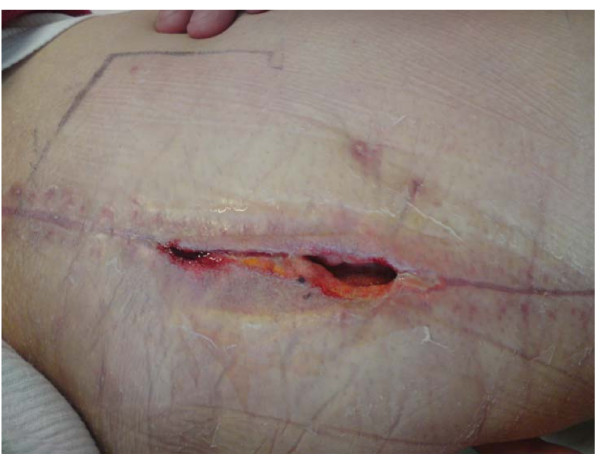
**Postoperative wound infection of the left upper limb six days after CABG operation with conventional saphenectomy**.

**Figure 2:**
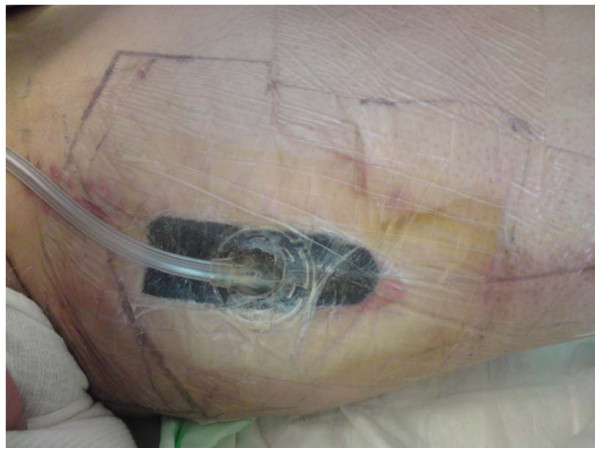
**Left upper limb treated with wound infection treated by a combination of vacuum-therapy and Daptomycin (Cubicin^®^, Novartis Pharma GmbH, Germany)**.

During the patient's stay in the ICU, hemodynamic conditions were stabilized and the patient was moved to a normal ward on second postoperative day. On 6^th ^postoperative day, after an uneventful mobilization, the patient was suffering from pain in his leg and he developed a wound infection of the left upper leg. Saphenctomy wound infection was presented with a large dehiscence of 5 cm in diameter in the location where the great saphenous vein had been harvested (Fig. 1). Clinical laboratory parameters (Leucocytes and CRP) were significantly increased and additionally to that, a wound swab was taken from the infected site and a gram-positive Staphylococcus epidermidis culture was indentified by the Institute of Microbiology and Hygiene of the University of Goettingen. The antibiogram presented methilin-resistence as well as resistence to several conventional antibiotics but displayed sensitivity to the new antibiotic drug Daptomycin (Cubicin^®^, Novartis Pharma Corporation, Germany). The drug was intravenously administered (Dose: 4 mg/kg body weight) and total duration of daptomycin-application was ten days. In addition, a VAC sponge was placed to low continuous wall suction in order to expedite the secondary wound healing process. During this time clinical labor parameters decreased significantly. Ten days later, another wound swab was taken from the dehiscent wound but no microbiological culture was identified. The wound was closed operatively and healed secondarily without any further complications (Fig. 2). An additional wound healing benefit was revealed by application by VAC therapy in combination with Daptomycin. The patient was discharged from hospital on the twenty-eighth postoperative day and was sent for further rehabilitative therapy.

**Figure 3:**
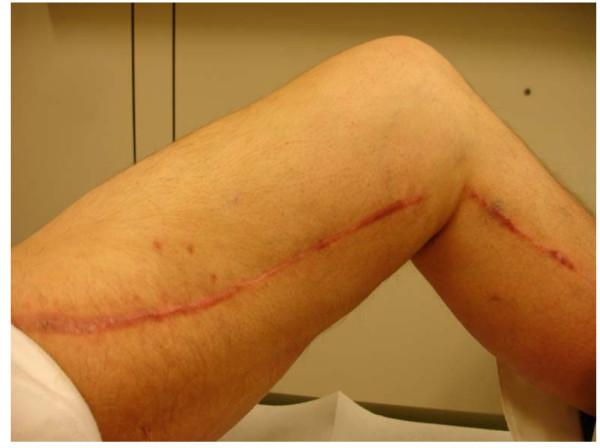
**Left upper limb after successful treatment of the wound infection by Daptomycin (Cubicin^®^, Novartis Pharma GmbH, Germany)**.

This case describes, to the best of our knowledge, the first successful treatment of a saphenectomy wound infection by the new antibiotic drug Daptomycin (Cubicin^®^, Novartis Pharma Corporation, Germany) (Fig. 3). Further research is required to evaluate the potential benefits of this new antibiotic agent. Also, clinical, prospective and randomized studies are necessary to compare several treatment methods for saphenectomy wound infections using different antibiotic drugs.

## Competing interests

The authors declare that they have no competing interests.

## Authors' contributions

JDS conceived of the study, and participated in its design and coordination. AFP conceived of the study, and participated in its design and coordination. STS conceived of the study, and participated in its design and coordination. KOC participated in the design of the study and performed the statistical analysis. SAM participated in the design of the study and performed the statistical analysis. AW participated in the design of the study and performed the statistical analysis. FAS conceived of the study, and participated in its design and coordination. Both JDS and AFP contributed equally to this study. All authors read and approved the final manuscript.

## Consent

Written informed consent was obtained from the patient for publication of this case report and accompanying images. A copy of the written consent is available for review by the Editor-in-Chief of this journal.
